# Supplementary material for: Arabidopsis species deploy distinct strategies to cope with drought stress
Source: Ann Bot. 2019 Jan 19;124(1):27–40. doi: 10.1093/aob/mcy237 (PMC6676377; doi:10.1093/aob/mcy237)
Supplement: mcy237_suppl_Supplementary_Material [file mcy237_suppl_supplementary_material.doc]

**Supplementary Information**

Article title: *Arabidopsis* species deploy distinct strategies to cope with drought stress

Authors: M. Bouzid, F. He, G. Schmitz, R.E. Häusler, A.P.M. Weber, T. Mettler-Altmann, J. de Meaux.

The following Supplementary Information is available for this article:

**Figure S1:** Summary of short read mapping to the *A. lyrata* reference genome V1.

**Figure S2:** Wilting day and soil moisture at wilting for the two first biological experiments of the drying-down experiments.

**Figure S3:** Soil water content during the first 7 days after water withdrawal.

**Figure S4:** Initial rosette area and leaf thickness of the plants used in the second biological experiments of the drying-down experiment.

**Figure S5:** Photosynthesis efficiency at wilting.

**Figure S6:** Proportion of surviving *A. halleri*, *A. lyrata*, and *A. thaliana* plants 2 days after re-watering for the two first biological experiments.

**Table S1**: List of accessions used for the dry-down experiments.

**Table S2:** Phenotypes measured in the three drying-down experiments.

**Table S3:** Phenotypic measurements including the three dry down experiments as well as stomata density and delta13C in standard conditions.

**Table S4**: Summary statistics of the multiple comparison of the wilting day between species.

**Table S5**: Summary statistics of the multiple comparison of the soil moisture at wilting between species.

**Table S6**: Summary statistics of the multiple comparison of the initial rosette area between species.

**Table S7**: Summary statistics of the multiple comparison of the initial leaf thickness between species.

**Table S8**: Summary statistics of the multiple comparison of the relative leaf water loss 7 days before wilting between species.

**Table S9**: Summary statistics of glm testing the effect of interaction between species and desiccation rate on the relative loss of leaf water content before wilting.

**Table S10**: Summary statistics of the multiple comparison of the photosynthetic efficiency at wilting between species.

**Table S11**: Summary statistics of the multiple comparison of the survival rate 2 days after re-watering between species.

**Table S12:** Differentially expressed genes identified for each of *Arabidopsis halleri* and *A. lyrata* between 20 and 60% of soil moisture and between recovery and 60% of soil moisture.

**Table S13:** Phenotypic data collected in this study. See methods for details on the measurements and experimental procedures.

**Figure S1:** Summary of short read mapping to the *A. lyrata* reference genome V1. Percentage of non-mapped reads at bottom, above that uniquely mapped, above that multiple mapped for each sample. Samples called ‘hal_1_c, hal_2_c, and hal_3_c’ are the three replicates of *A. halleri* plants sampled at 60% of soil moisture; ‘hal_1_t, hal_2_t, and hal_3_t’ are the three replicates of *A. halleri* plants sampled at 20-25% of soil moisture; ‘hal_1_r, hal_2_r, and hal_3_r’ are the three replicates of *A. halleri* plants sampled after recovery. The same is for *A. lyrata* samples ‘lyr’.


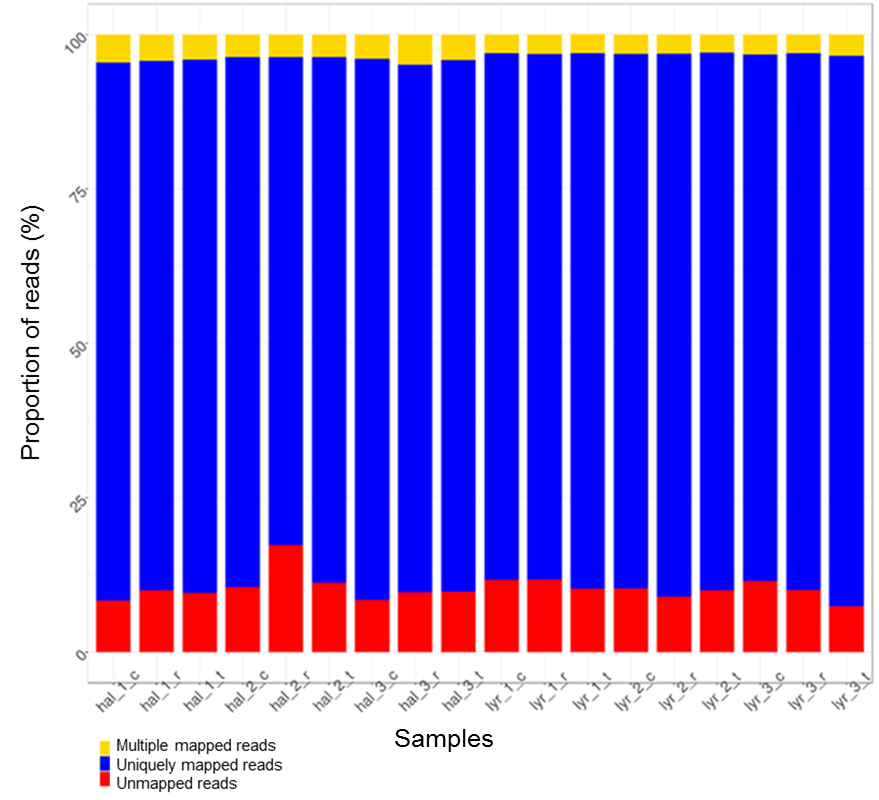


**Figure S2:** Wilting day and soil moisture at wilting for the two first biological experiments of the drying-down experiments. ***(a)*** Number of days between initiation of soil dry down treatment and wilting. ***(b)*** Soil moisture at wilting for *Arabidopsis halleri*, *A. lyrata*, and *A. thaliana*. Letters above violin plots indicate significant differences between species (*Tukey’s HSD test, P value <0.05*). Results are shown for the first two biological experiments.

**
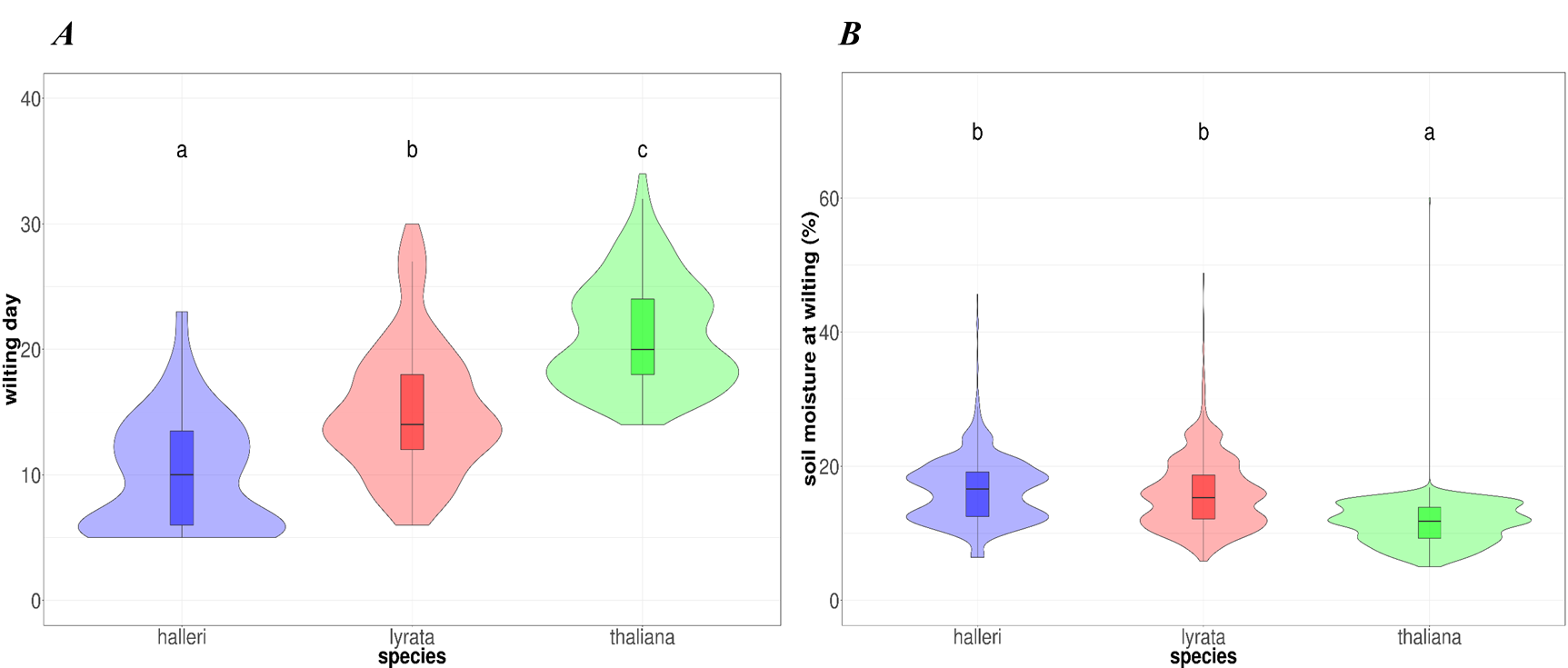
**

**Figure S3:** Soil water content during the first 7 days after water withdrawal. Decrease in soil water content after water withdrawal in the first ***(a)*** and the second ***(b)*** biological experiments for *Arabidopsis halleri*, *A. lyrata*, and *A. thaliana*. Shaded ribbons represent the standard deviation. P-values show the significant interaction between time and species effect on the water content of soil.


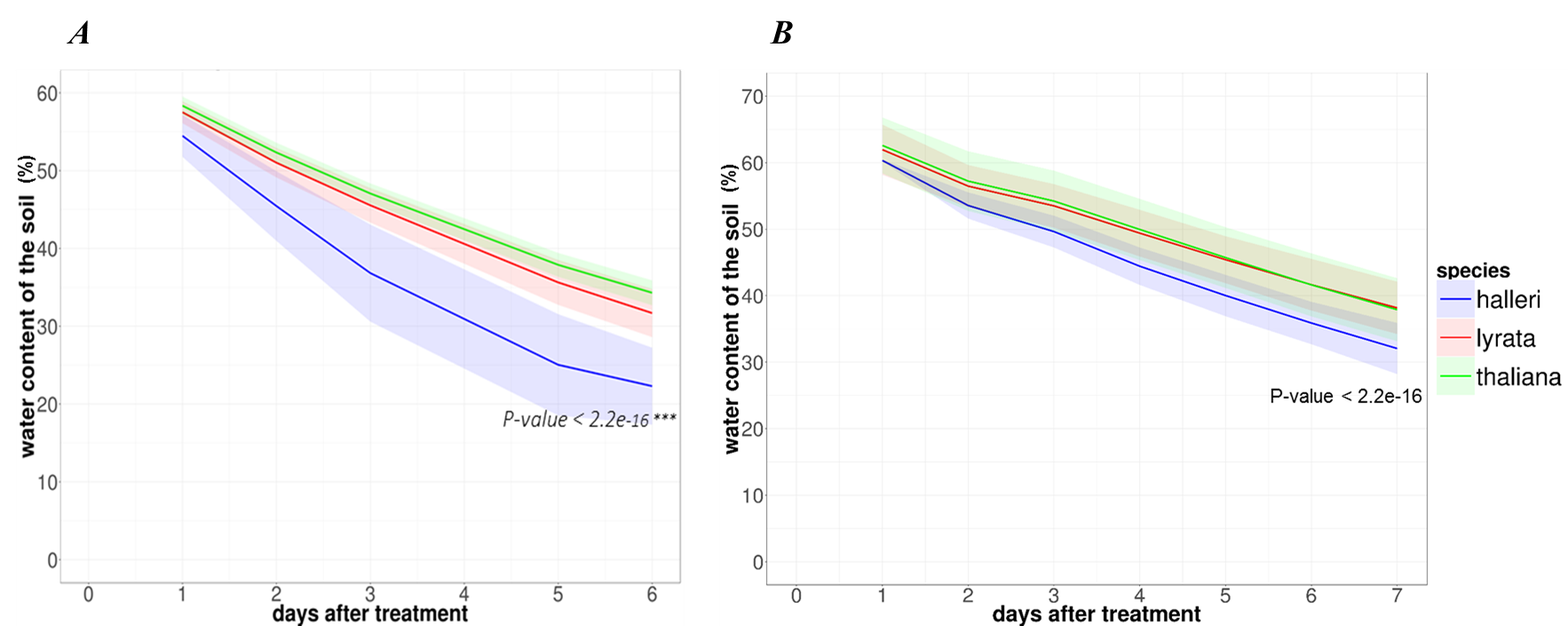


**Figure S4:**  Initial rosette area and leaf thickness of the plants used in the second biological experiment of the drying-down experiment. ***(a)*** Rosette leaf area (mm²) and ***(b)*** initial leaf thickness measured (mm) at 60% of soil moisture (before water withdrawal). Data were collected in the second biological experiment for *Arabidopsis halleri*, *A. lyrata*, and *A. thaliana*. Box plots with the same letter are not significantly different (*Tukey’s HSD, P value <0.05*).

**
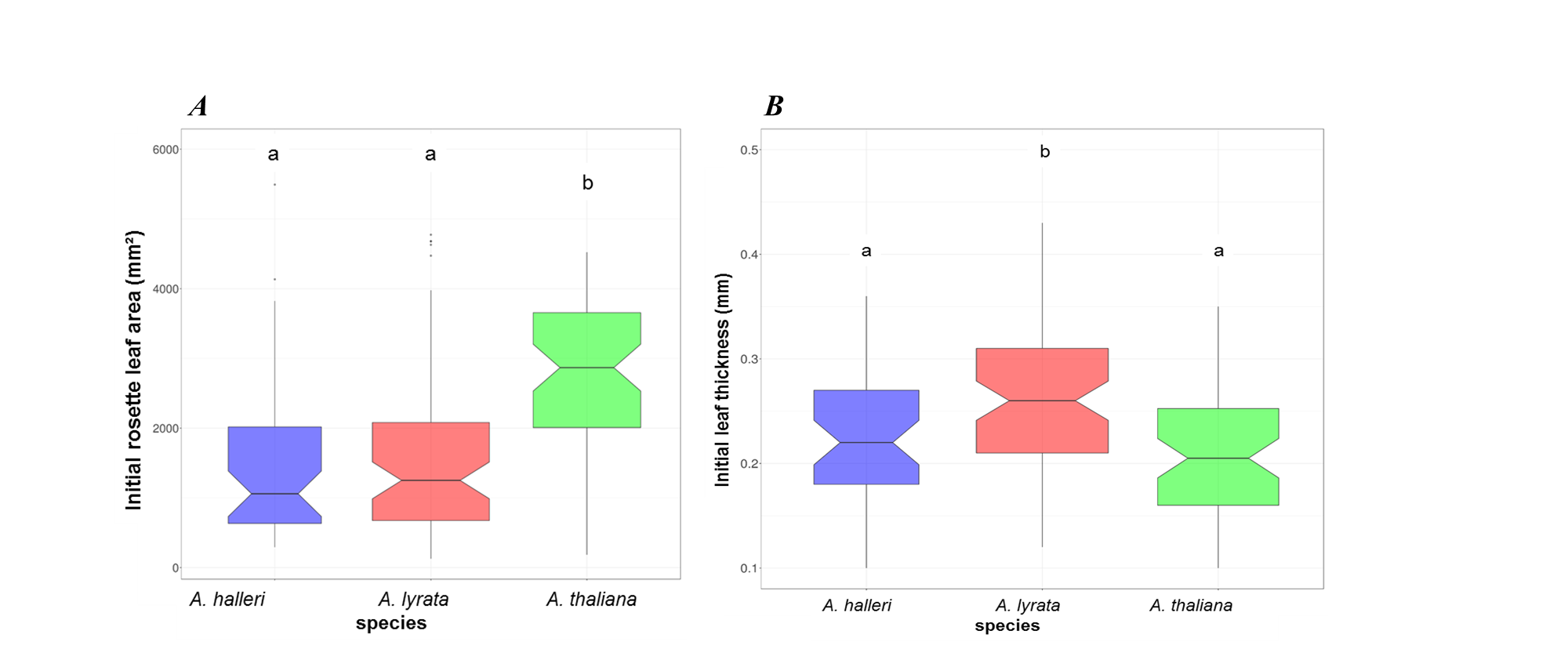
**

**Figure S5:** Photosynthesis efficiency at wilting. Percentage of maximum photosystem II efficiencies at wilting compared to the initial efficiencies. The average initial F_v_ : F_m_ ratios and the standard deviation for *A. halleri*, *A lyrata*, and *A. thaliana* were: 0.735 ± 0.11; 0.76 ± 0.052; 0.77 ± 0.008 respectively. Violin plots with the same letter are not significantly different according to Tukey’s HSD (P value <0.05). Results are shown for the first biological experiment.


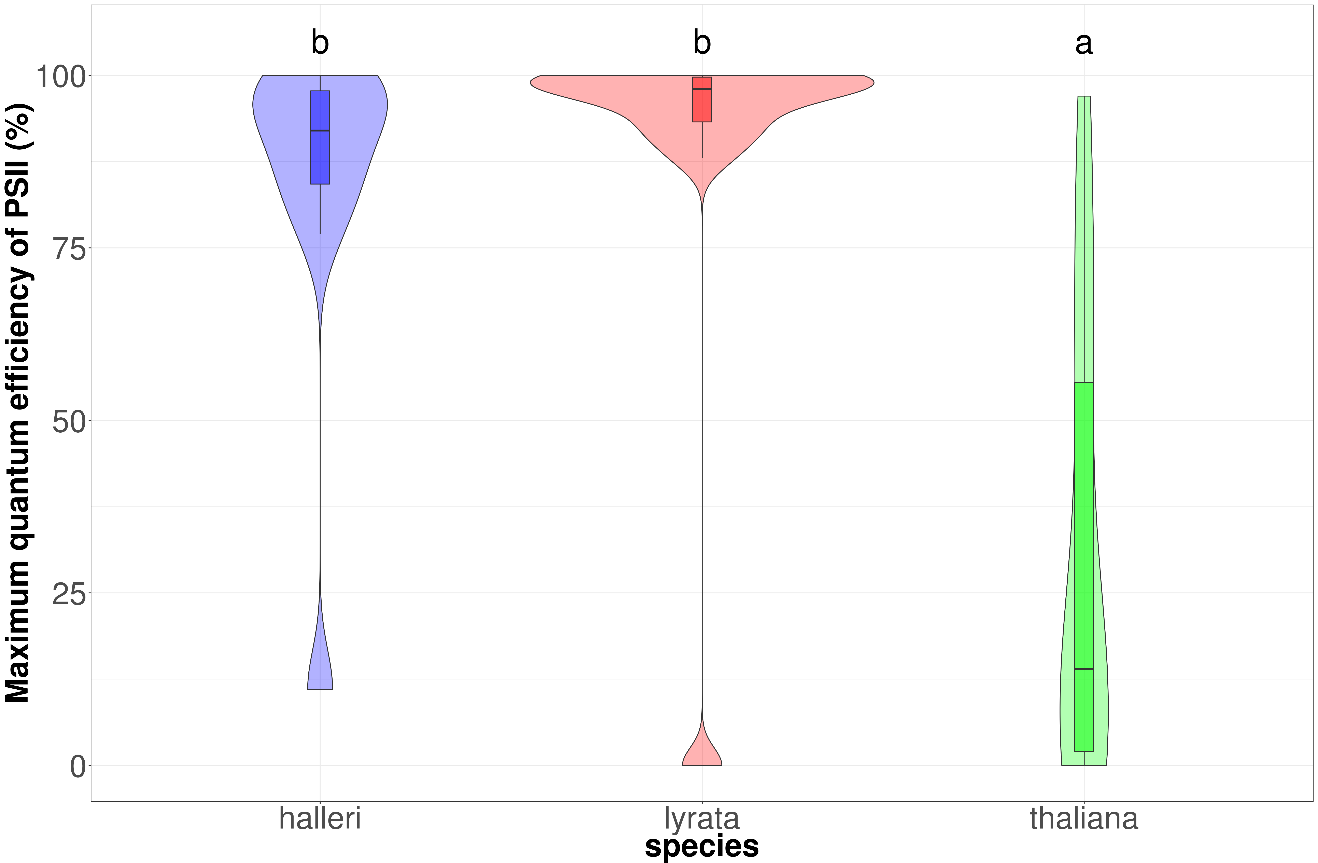


**Figure S6:** Proportion of surviving *A. halleri*, *A lyrata*, and *A. thaliana* plants 2 days after re-watering for the two first biological experiments. Letters above violin plots indicate significant differences between species (*Tukey’s HSD test, P value <0.05*). Results are shown for the two first biological experiments.


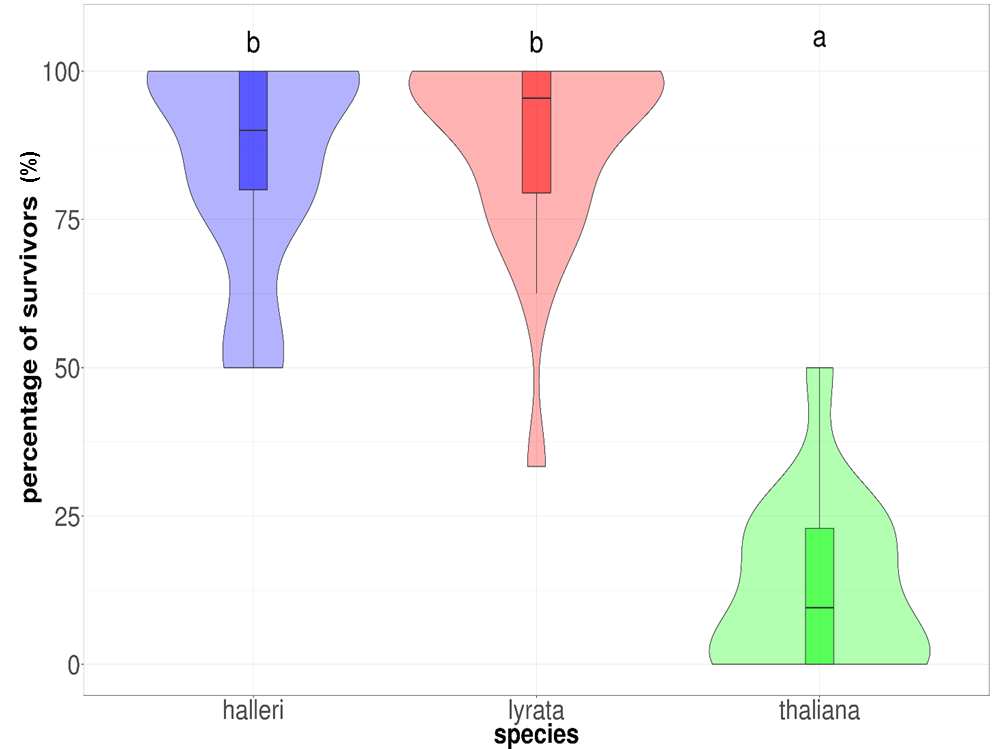


**Table S1**: List of accessions used for the dry-down experiments.

| Species | accessions | Country | Latitude | Longitude |
| --- | --- | --- | --- | --- |
| *A. lyrata* | SB12 | Germany | 51.31 | 10.55 |
| *A. lyrata* | LF2 | Austria | 47.59 | 15.36 |
| *A. lyrata* | LF10 | Austria | 47.59 | 15.36 |
| *A. lyrata* | NT12 | Germany | 49.31 | 11.32 |
| *A. lyrata* | Vos | Austria | 47.58 | 16.10 |
| *A. lyrata* | Plech91.4a | Germany | 49.37 | 11.30 |
| *A. lyrata* | PlechC3 | Germany | 49.37 | 11.30 |
| *A. lyrata* | Plech61.2a | Germany | 49.37 | 11.30 |
| *A. lyrata* | Plech92.2a | Germany | 49.37 | 11.30 |
| *A. lyrata* | Plech.Rock79b | Germany | 49.37 | 11.30 |
| *A. lyrata* | Plech73.3a | Germany | 49.37 | 11.30 |
| *A. lyrata* | Plech85.1a | Germany | 49.37 | 11.30 |
| *A. lyrata* | Plech61.4a | Germany | 49.37 | 11.30 |
| *A. lyrata* | Plech.61.a19 | Germany | 49.37 | 11.30 |
| *A. lyrata* | HAS.166b | Germany | NA | NA |
| *A. lyrata* | HAS.005 | Germany | NA | NA |
| *A. lyrata* | HAS.122c | Germany | NA | NA |
| *A. lyrata* | HAS120 | Germany | NA | NA |
| *A. lyrata* | HAS101c | Germany | NA | NA |
| *A. lyrata* | HAS114 | Germany | NA | NA |
| *A. lyrata* | MN47 | US, Michigan | NA | NA |
| *A. lyrata* | Sky | Scotland | NA | NA |
| *A. halleri* | Laut3 | Germany | 51.86 | 10.30 |
| *A. halleri* | Laut11 | Germany | 51.86 | 10.30 |
| *A. halleri* | Wall7 | Germany | 50.41 | 11.56 |
| *A. halleri* | Wall10 | Germany | 50.41 | 11.56 |
| *A. halleri* | Lita6 | Czech | 49.77 | 14.01 |
| *A. halleri* | Kowa7 | Poland | 50.76 | 15.85 |
| *A. halleri* | Krom10 | Slovakia | 48.92 | 20.90 |
| *A. halleri* | Bara4 | Romania | 47.69 | 23.63 |
| *A. halleri* | Bara3 | Romania | 47.69 | 23.63 |
| *A. halleri* | Nisu6 | Romania | 46.86 | 22.81 |
| *A. halleri* | Nisu5 | Romania | 46.86 | 22.81 |
| *A. halleri* | Prev2 | Slovenia | 46.52 | 15.52 |
| *A. halleri* | Prev6 | Slovenia | 46.52 | 15.52 |
| *A. halleri* | Lobn5 | Slovenia | 46.52 | 15.52 |
| *A. halleri* | Lobn6 | Slovenia | 46.52 | 15.52 |
| *A. halleri* | Noss10 | Italy | 45.86 | 9.88 |
| *A. halleri* | hal2.2 | Italy | 45.86 | 9.84 |
| *A. thaliana* | IP-Ara-4 | Spain | 41.70 | -3.68 |
| *A. thaliana* | IP-Cmo-3 | Spain | 40.05 | -4.65 |
| *A. thaliana* | IP-Hom-4 | Spain | 40.82 | -1.68 |
| *A. thaliana* | IP-Lab7 | Spain | 40.40 | -5.00 |
| *A. thaliana* | Amu-0 | Spain | 40.87 | -4.50 |
| *A. thaliana* | Coy-0 | Spain | 40.44 | -4.27 |
| *A. thaliana* | Gud-3 | Spain | 40.65 | -4.11 |
| *A. thaliana* | Hec-0 | Spain | 42.86 | -0.70 |
| *A. thaliana* | Hue-3 | Spain | 42.96 | -6.10 |
| *A. thaliana* | Pdl-0 | Spain | 43.02 | -5.60 |
| *A. thaliana* | Prd-0 | Spain | 41.14 | -3.68 |
| *A. thaliana* | Som-0 | Spain | 41.14 | -3.58 |
| *A. thaliana* | Urd-1 | Spain | 42.27 | -2.98 |
| *A. thaliana* | Val-0 | Spain | 42.31 | -3.10 |
| *A. thaliana* | Col-Fri | NA | NA | NA |

**Table S2:** Phenotypes measured in the three drying-down experiments.

| **Trait** | **Experiment 1** | **Experiment 2** | **Experiment 3** | |
| --- | --- | --- | --- | --- |
| **Soil moisture** | ✓ | ✓ | ✓ |  |
| **Wilting day** | ✓ | ✓ |  |  |
| **Leaf thickness** |  | ✓ |  |  |
| **Initial rosette area** |  | ✓ |  |  |
| **Photosynthesis** | ✓ |  |  |  |
| **Survival rate** | ✓ | ✓ |  |  |
| **Drought damage rate** |  | ✓ |  |  |
| **Transcript abundance** |  |  | ✓ |  |

**Table S3:** Number of accessions used in the three drying-down experiments.

| **Number of accessions** | ***A. halleri*** | ***A. lyrata*** | ***A. thaliana*** |
| --- | --- | --- | --- |
| **Experiment 1** | 13 | 22 | 16 |
| **Experiment 2** | 12 | 16 | 12 |
| **Experiment 3** | 10 | 8 | 10 |

**Table S4**: Summary statistics of the multiple comparison of the wilting day between species. Simultaneous tests for general linear hypotheses, multiple comparison of means: Tukey contrast; fit: glm (formula = wilting_day ~ species + experiments, family = negative binomial (theta = 130041))

| *Linear Hypotheses* | *Estimate* | *Std. Error* | *z value* | *Pr(>|z|)* |
| --- | --- | --- | --- | --- |
| *lyrata - halleri == 0* | 0. 09238 | 0. 01162 | 7.95 | <1e-10 |
| *thaliana - halleri == 0* | -0. 15550 | 0. 01218 | -12.77 | <1e-10 |
| *thaliana - lyrata == 0* | -0. 24788 | 0. 01249 | -19.84 | <1e-10 |

**Table S5**: Summary statistics of the multiple comparison of the soil moisture at wilting between species. Simultaneous tests for general linear hypotheses, multiple comparison of means: Tukey contrast; fit: glm (formula = soil moisture ~ species + experiments, family = negative binomial (theta = 5.14))

| *Linear Hypotheses* | *Estimate* | *Std. Error* | *z value* | *Pr(>|z|)* |
| --- | --- | --- | --- | --- |
| *lyrata - halleri == 0* | 0. 01929 | 0. 03639 | 0.530 | 0.856 |
| *thaliana - halleri == 0* | -0. 27688 | 0. 03638 | -7.611 | <1e-10 |
| *thaliana - lyrata == 0* | -0. 29616 | 0. 03851 | -7. 691 | <1e-10 |

**Table S6**: Summary statistics of the multiple comparison of the initial rosette area between species. Simultaneous tests for general linear hypotheses, multiple comparison of means: Tukey contrast; fit: glm (formula = initial rosette area ~ species, family = negative binomial (theta = 5.14))

| *Linear Hypotheses* | *Estimate* | *Std. Error* | *z value* | *Pr(>|z|)* |
| --- | --- | --- | --- | --- |
| *thaliana - halleri == 0* | 0. 64040 | 0. 15940 | 4.017 | 0. 000175*** |
| *lyrata - halleri == 0* | 0. 07326 | 0. 15402 | 0.476 | 0. 882542 |
| *lyrata - thaliana == 0* | -0. 56714 | 0. 14175 | -4. 001 | 0.000181 *** |

**Table S7**: Summary statistics of the multiple comparison of the initial leaf thickness between species. Simultaneous tests for general linear hypotheses, multiple comparison of means: Tukey contrast; fit: glm (formula = initial leaf thickness ~ species, family = negative binomial (theta = 194918))

| *Linear Hypotheses* | *Estimate* | *Std. Error* | *z value* | *Pr(>|z|)* |
| --- | --- | --- | --- | --- |
| *lyrata - halleri == 0* | 0.13339 | 0.05566 | 2.396 | 0.0435 * |
| *thaliana - halleri == 0* | -0.07336 | 0.06001 | -1.222 | 0.4389 |
| *thaliana - lyrata == 0* | -0.20675 | 0.05210 | -3.969 | <0.001 *** |

Signif. codes: 0 ‘***’ 0.001 ‘**’ 0.01 ‘*’ 0.05 ‘.’ 0.1 ‘ ’ 1

**Table S8**: Summary statistics of the multiple comparison of the relative leaf water loss 7 days before wilting between species. Simultaneous tests for general linear hypotheses, multiple comparison of means: Tukey contrast; fit: glm (formula = ratio leaf thickness 2 by 7 days before wilting ~ species, family = negative binomial (theta = 212261))

| *Linear Hypotheses* | *Estimate* | *Std. Error* | *z value* | *Pr(>|z|)* |
| --- | --- | --- | --- | --- |
| *lyrata - halleri == 0* | 0.13113 | 0. 05342 | 2. 455 | 0. 0372 * |
| *thaliana - halleri == 0* | -0. 04045 | 0. 05758 | -0. 703 | 0. 7613 |
| *thaliana - lyrata == 0* | -0. 17159 | 0. 05001 | -3. 431 | 0. 0018 ** |

**Table S9**: Summary statistics of glm testing the effect of interaction between species and desiccation rate on the relative loss of leaf water content before wilting.

Model: glm (formula = ratio leaf thickness 2 by 7 days before wilting ~ species * desiccation rate, family = negative.binomial (theta = 221492))

| *Coefficients* | *Estimate* | *Std. Error* | *t value* | *Pr(>|t|)* |
| --- | --- | --- | --- | --- |
| *Intercept* | -0.458698 | 0.140235 | -3.271 | 0.001117 ** |
| *Species lyrata* | 0.200251 | 0.180193 | 1.111 | 0.000137 *** |
| *Species halleri* | -0.815855 | 0.212890 | -3.832 | 0.000137 *** |
| *Dessication rate* | -0.013724 | 0.034552 | -0.397 | 0.691320 |
| *Species lyrata:dessication rate* | 0.0058098 | 0.044901 | 0.114 | 0.909623 |
| *Species halleri:dessication rate* | -0207574 | 0.051474 | -4.033 | 6.03e-05 *** |
| *Species thaliana: dessication rate* | -0.005098 | 0.044901 | -0.114 | 0.9096 |

Dispersion parameter for Negative Binomial (221492) family taken to be 0.05412319

Null deviance: 401.31 on 823 degrees of freedom

Residual deviance: 395.58 on 818 degrees of freedom

AIC: 1460.3

Number of Fisher Scoring iterations: 4

**Table S10**: Summary statistics of the multiple comparison of the photosynthetic efficiency at wilting between species. Simultaneous tests for general linear hypotheses, multiple comparison of means: Tukey contrast; fit: glm (formula = photosynthetic capacity at wilting ~ species, family =quasi-poisson)

| *Linear Hypotheses* | *Estimate* | *Std. Error* | *z value* | *Pr(>|z|)* |
| --- | --- | --- | --- | --- |
| *lyrata - halleri == 0* | 0.07337 | 0.30653 | 0.239 | 0.96885 |
| *thaliana - halleri == 0* | -1.03034 | 0.30362 | -3.394 | 0.001998 * |
| *thaliana - lyrata == 0* | -1.10371 | 0.27656 | -3.991 | 0.000187*** |

**Table S11**: Summary statistics of the multiple comparison of the survival rate 2 days after re-watering between species. Simultaneous tests for general linear hypotheses, multiple comparison of means: Tukey contrast; Fit: glm (formula = survival ~ species, family =quasi-binomial)

| *Linear Hypotheses* | *Estimate* | *Std. Error* | *z value* | *Pr(>|z|)* |
| --- | --- | --- | --- | --- |
| *lyrata - halleri == 0* | -0.01762 | 0.05674 | -0.311 | 0.948 |
| *thaliana - halleri == 0* | -0.77383 | 0.06023 | -12.847 | <1e-06 *** |
| *thaliana - lyrata == 0* | -0.75621 | 0.05415 | -13.966 | <1e-06 *** |
